# Incidence of Aortic Regurgitation in Association with Type of Ventricular Septal Defects and its Immediate and Intermediate Outcome after Surgical Closure

**DOI:** 10.7759/cureus.5102

**Published:** 2019-07-08

**Authors:** Veena Kumari, Abdul S Shaikh, Saad B Zakai, Naresh Kumar, Sohail K Bangash, Najma Patel

**Affiliations:** 1 Paediatric Cardiology, National Institute of Cardiovascular Diseases, Karachi, PAK; 2 Paediatric Cardiac Surgery, National Institute of Cardiovascular Diseases, Karachi, PAK

**Keywords:** aortic regurgitation, vsd, surgical vsd closure, residual vsd

## Abstract

Introduction

Ventricular septal defect (VSD) is one of the more common congenital heart defects, and aortic regurgitation (AR) is its major complication if it remains unrepaired. We aim to determine the AR incidence in various types of VSD, its immediate and intermediate six to 12-month post-VSD repair outcomes of AR.

Methods

We conducted a retrospective review of medical records of all children aged 18 years or younger who were diagnosed with single VSD at our institution from 2016 to 2018. VSD was classified according to its location and relation to the tricuspid annulus and semilunar valve. AR severity grading was done according to the American Society of Echocardiography, and vena contracta width (VC) was taken as the main parameter for severity. We defined trivial-to-mild AR as VC width less than 0.3 cm, moderate AR was 0.3-0.6 cm VC width, and severe AR was VC width of more than 0.6 cm. Immediate and intermediate outcomes of surgical closure, such as residual VSD and AR, were observed.

Results

One hundred ninety patients with isolated single VSD were included in the study. Of those, 114 patients had perimembranous VSD (60.0%), 64 patients had muscular VSD (33.7%), and 12 patients had supracristal VSD (6.3%). The median age of our study cohort was six months, with a male to female ratio of 1.3:1. Aortic valve prolapse (28.9%; n = 55) and AR (23.2%; n = 44) were the most common findings on echocardiographic evaluation of VSD patients. Most cases of VSD with AR had trivial-to-mild AR, (68.2%; n = 30). AR was most commonly seen in supracristal VSD (83.3%; n = 10) followed by perimembranous VSD (28.9%; n = 33). VSD closed spontaneously in 34 patients (17.9%) and 98 patients (51.6%) patients underwent surgery. Residual VSD after surgical closure was present in 57.1% (56) and 17.3% (17) of the patients immediate postoperatively and six- to 12-month postoperative follow-up, respectively. Similarly, residual AR after surgical closure of VSD was present in 32.7% (32) and 15.3% (15) of the patients immediate postoperatively and six- to 12-month postoperative follow-up, respectively.

Conclusion

The incidence of AR with VSD was very high in our study; AR was most commonly associated with supracristal VSD. After surgical repair, mild AR decreased with time. Early corrective surgery of VSD can prevent this complication and help improve outcomes.

## Introduction

Of all congenital heart disease (CHD), ventricular septal defects (VSDs) are the most common form [1-2]. VSDs result from a deficiency or a failure of alignment or fusion of components of the ventricular septum during the embryological development of the heart [3].

The natural history of an untreated or undiagnosed VSD that does not spontaneously close is associated with several complications like hemodynamic disturbances, congested cardiac failure, growth failure, right ventricular or left ventricular outflow tract obstruction, aortic valve prolapse (AVP), and subsequent development of aortic regurgitation (AR) [4-5]. AR is the important and major complication, and the risk of AVP and AR progressively increases if VSD left untreated [4]. These factors ultimately increase the mortality associated with unrepaired VSD. Spontaneous VSD closure usually occurs in the first year of life [2]. Furthermore, the first two years of life are considered crucial in terms of early surgical correction, which leads to higher chances of complete recovery of left ventricular function. Given that these defects rarely close after age two, early and correct identification of the type and size of VSD is important for positive outcomes [5].

The incidence of CHD in Pakistan is highly underestimated due to the popular practices of home deliveries and quick discharge of neonates from hospital deliveries. VSDs are estimated to be the most common cardiac defects in Pakistan, responsible for 25%-45% of all congenital cardiac defects according to some hospital-based studies [6-9].

VSDs are divided into three types: perimembranous (pm), muscular, and doubly committed subarterial (DCSA)/Supracristal [10]. Although the development of complications from a VSD depends on its size, the type of defect also dictates its natural history. pm-VSDs are more common, but muscular VSDs have a better outcome; their spontaneous closure takes place earlier as compared to pm-VSDs. DCSA-VSDs have poor outcomes due to their association with the progressive development of AVP and AR [5,11-12].

Regarding the type of VSDs, the limited data from Pakistan show slightly contrasting results. One study showed DCSA as the least common type of VSD, and another study showed muscular VSDs as the least common; both, however, concluded pm-VSD as the most common [7]. AR is progressive, and the presence of even AVP or mild AR is an indication for surgery [13-14]. In our country, many VSDs remain uncorrected or present at a later age, so it would be interesting to see the incidence of AVP and AR in our patients along with the outcome after surgical VSD repair. These data may help to encourage physicians to correct VSD early to decrease the incidence of complications such as AVP and AR.

## Materials and methods

We retrospectively reviewed the medical records and echocardiographic findings of all patients aged 18 or younger with a single VSD diagnosis via transthoracic echocardiography at our institution from 2016 to 2018. Approval of the ethical review committee was obtained (ERC-27/2019). The study included children who had at least one presurgical echocardiographic study and two postsurgical studies (within 24 to 48 hours and six to 12 months postoperative). Children were excluded if they had complex congenital heart disease or multiple VSDs or VSD with any other associated CHD and if they died immediately postoperative. The study ultimately included 190 patients. VSDs were classified according to location and relation to the tricuspid annulus and semilunar valve, as described by Sutherland et al. [15]. Aortic valve prolapse (AVP) was diagnosed when a deformed aortic cusp pivoted from the crest of the interventricular septum, which is noticeable in a parasternal long-axis view [16].

AR severity grading was done according to the American Society of Echocardiography, and vena contracta width (VC) was taken as the main parameter for severity. AR was defined as trace-to-mild if VC width was less than 0.3 cm, moderate AR was defined as VC width of 0.3 cm to 0.6 cm, and severe AR was defined as VC width more than 0.6 cm [17].

Statistical analysis was performed by using IBM SPSS Statistics for Windows, Version 21.0. (IBM Corp., Armonk, NY, US). Results were presented as mean and standard deviation for continuous variables (i.e., patient age and size of VSD) and frequency and percentage for categorical variables (i.e., gender, AR, type of VSD, and severity of AR). The chi-square test was applied, and a p-value of ≤0.05 was considered significant.

## Results

pm-VSD was the most common type (60%) in our study population. AVP was noted in 55 patients (28.9%), while AR was noted in 44 (23.2%) patients. Demographic characteristics of the patients, types of VSD, number of spontaneously closed VSDs, and number of patients who underwent surgery with residual VSD or AR are shown in Table 1.

**Table 1:** Demographic profile and postoperative outcomes of the study population Abbreviations: AR, aortic regurgitation; AVP, aortic valve prolapse; CHF, congestive heart failure; DCSA, doubly committed subarterial; VSD, ventricular septal defect; SD, standard deviation; IQR, interquartile range.

Demographic feature and outcomes	n = 190
Age in months (median and IQR)	6 (3-20)
Percent of males (n)	55.8% (106)
Mean VSD size ± SD	5.0 mm ± 2.7 mm
Type of VSD	
Perimembranous, conoventricular (n)	60% (114)
Supracristal / DCSA, conotruncal (n)	6.3% (12)
Muscular (n)	33.7% (64)
AVP (n)	28.9% (55)
AR (n)	23.2% (44)
Sign and symptoms of CHF (n)	51.1% (97)
Associated dysmorphic features/ syndrome (n)	18.9% (36)
Spontaneously closure (n)	17.9% (34)
No indication for surgery (n)	30.5% (58)
Underwent surgery (n)	51.6% (98)
Residual VSD (n = 98)	
Immediate postoperatively (n)	57.1% (56)
Within one year follow-up(n)	17.3% (17)
Residual AR [n = 98]	
Immediate postoperatively (n)	32.7% (32)
Within one year follow-up (n)	15.3% (15)

Patients with supracristal VSD had the highest incidence of AR (83.3%; n = 10/12), and patients with muscular VSD had the lowest incidence of AR (1.6%; n = 1/64), as shown in Table 2.

**Table 2:** Frequency of aortic regurgitation according to the type of ventricular septal defect preoperatively Abbreviation: VSD, ventricular septal defect. ^a^P-value is based on chi-square test *Significant at 5%

Type of VSD	n	Aortic regurgitation		
		Absent	Present	p-Value^a^
Perimembranous	114	71.1% (81)	28.9% (33)	<0.001*
Supracristal	12	16.7% (2)	83.3% (10)	
Muscular	64	98.4% (63)	1.6% (1)	

Among the patients with preoperative AR, most of the patient had trivial-to-mild AR (68.2%; n = 30), as shown in Table 3. Of the 190 total children, 98 patients (51.6%) underwent surgery, and residual VSD was found in 56 patients (57.1%) in the immediate postoperative period. The incidence of residual VSD reduced to 17.3% (n = 17) after the one-year follow-up (p≤.001) as shown in Table 3. Immediate postoperative residual AR was observed in 32 children (32.7%), a reduction from the preoperative frequency of AR (44.9%; n = 44; p = 0.079). At the one-year follow-up evaluation, the incidence was further reduced (15.3%; n = 15; p≤.001).

**Table 3:** Residual ventricular septal defect, aortic regurgitation and its severity grading, preoperatively, immediate postoperatively, and within one-year follow-up in patients receiving surgical ventricular septal defect closure Abbreviations: AR, aortic regurgitation; VSD, ventricular septal defect. *Based on patients with residual VSD **Based on patients with AR

Outcomes	Preoperative status	Immediate postoperatively	Within one year postoperatively
N	98	98	98
Residual VSD (n)	-	57.1% (56)	17.3% (17)
*Size of Residual VSD			
<2 mm (n)	-	69.6% (39)	47.1% (8)
>2 mm (n)	-	30.4% (17)	52.9% (9)
AR (n)	44.9% (44)	32.7% (32)	15.3% (15)
**AR Severity Grading			
Trivial-to-Mild (n)	68.2% (30)	71.9% (23)	66.7% (10)
Moderate (n)	25% (11)	25% (8)	33.3% (5)
Severe (n)	6.8% (3)	3.1% (1)	0% (0)

AR severity also improved; of the 30 children who had trivial-to-mild AR preoperatively, 23 retained it in the immediate postoperative period, and 10 still had it at the six- to 12-month follow-up. Of the 11 children with moderate AR who needed aortic valve repair, eight children still had AR in the immediate postoperative period, and five had AR at the six- to twelve-month follow-up. All patients with severe AR underwent aortic valve replacement. One of them had severe AR as an immediate complication due to paravalvular leakage for which reoperation was performed. None had severe AR at the 12-month follow-up (Table 3).

## Discussion

Percentage of spontaneous closure of VSDs is variable and depends upon the age of the patient, the type and size of the VSD. The literature indicates a wide range of 8.8% to 85% of cases spontaneously close [2,18]. In our study, spontaneous closure occurred in 17.9% of cases. The natural history of VSD shows that spontaneous closure rarely occurs after the first two years of life and is associated with many complications, the most important of which is AR [5,7]. Once AR develops, its progressive nature is associated with significant mortality, and it becomes highly unlikely that VSD will close spontaneously. This natural history suggests early surgical intervention [19-20].

Data from Pakistan are scarce regarding the incidence of AR with VSD. Few studies exist on the incidence of VSD, frequency of its types, early repair, and outcomes [6-8]. In our study, pm-VSD was the most common (60%), which aligns with several Western studies [20-21]. Eroglu et al. reported an incidence of pm-VSD of 65% and DCSA of 12% [20]. Aziz et al. reported a pm-VSD incidence of 92%, whereas DCSA-VSD incidence was only 7% [21]. Studies from Japan and China report the incidence of DCSA-VSD to be 30% [22].

According to our findings, muscular VSD was the second-most common with a frequency of 34%, while DCSA was the least common at 6%. The median age of patients in our study was six months, which is similar to other studies in Pakistan by Aziz et al. [21], Kazmi et al. [7], and Chaudhry et al. [23].

A subset of patients with VSD develop AR; these may be pm or DCSA. The literature has shown that AR implicates DCSA more than pm-VSD. The mechanisms responsible for AVP are a lack of leaflet structural support due to VSD, abnormalities in commissural suspension, a lack of oppositional forces, a lack of continuity between the aortic media and annulus, and the venturi effect [14]. Tohyama et al. reported that AVP developed in 69% of cases with DCSA-VSD [16]. Most of the literature from Western countries report a higher incidence of AR with DCSA. However, data published from Pakistan show a higher incidence of AR in cases of pm-VSD [7,23]. Contrary to local studies, our population showed a higher incidence of AR in DCSA-VSD (83.3%) than pm-VSD (28.9%; p<0.001).

One of the indications for the closure of VSD in both DCSA and pm-VSD is the presence of AR and AVP. Even if the AR is trivial, it is still progressive. Lun et al. proposed that all DSCA-VSD ≥ 5 mm should be closed regardless of the development of AVP or AR [24]. Closure of VSD before the development of cusp deformation will prevent the development of AR. The incidence of postoperative residual VSD varies according to the type and size of VSD being repaired. Malaligned and muscular VSD repair may produce higher residual rates [25-26]. Sayadpour-Zanjani et al. reported postoperative residual VSD in 56% of cases [27]. Residual shunts may result in left ventricular volume overload, pulmonary arterial hypertension, and risk of bacterial endocarditis. Residual shunts have an impact on the postoperative recovery period and morbidity of the patient in addition to the financial and psychological status of the parents or guardians. Patients with residual VSDs need continued endocarditis prophylaxis and periodic follow-up to determine the need for repeated surgery. A Qp/Qs>1.5:1 is an indication for re-intervention [25-26,28].

Ninety-eight (51.6%) patients in our study underwent surgical closure, and 56 (57.1%) had a residual VSD immediately following the procedure. Of those, 39 cases (69.6%) of residual VSD closed spontaneously within one year and remained patent in 17 (30.4%) patients. Bol-Raap et al. [29] reported spontaneous closure of residual VSDs in 51% during the follow-up period of 3.9 years; they describe a high rate of spontaneous closure if the residual shunt is small [29]. The same trend was observed in our study, as children who had residual defects <2 mm had a high rate of spontaneous closure within one year postoperatively (31/39; 80%) compared to those with defects >2 mm (8/17; 47%; p = 0.028).

The reduced AR due to surgical valve repair we noted in our study signifies the beneficial effects of timely surgery on chances of complete resolution of AR. AR resolves or improves in up to 84% of patients postoperatively, and younger patients tend to experience better results [22,30].

Our study was limited by its retrospective nature, and we did not review the original echocardiogram findings. Our study was also limited in that we used a short follow-up period of one year.

## Conclusions

While pm-VSD is the most common type of VSD, DCSA/supracristal is most important due to its association with the development of AR. Postoperatively, AR decreases in severity and may completely disappear. Early surgical correction is advisable to prevent the occurrence and progression of AR to reduce morbidity and mortality associated with VSD complications.
